# HPV DNA Associates With Breast Cancer Malignancy and It Is Transferred to Breast Cancer Stromal Cells by Extracellular Vesicles

**DOI:** 10.3389/fonc.2019.00860

**Published:** 2019-09-16

**Authors:** Sabrina De Carolis, Gianluca Storci, Claudio Ceccarelli, Claudia Savini, Lara Gallucci, Pasquale Sansone, Donatella Santini, Renato Seracchioli, Mario Taffurelli, Francesco Fabbri, Fabrizio Romani, Gaetano Compagnone, Cristina Giuliani, Paolo Garagnani, Massimiliano Bonafè, Monica Cricca

**Affiliations:** ^1^Department of Experimental, Diagnostic and Specialty Medicine, University of Bologna, Bologna, Italy; ^2^Center of Applied Biomedical Research (CRBA), S. Orsola-Malpighi Hospital, Bologna, Italy; ^3^Department of Medicine, Memorial Sloan Kettering Cancer Center, New York, NY, United States; ^4^Department of Infectious Diseases, Integrative Virology, CIID, University Hospital Heidelberg, Heidelberg, Germany; ^5^Children's Cancer and Blood Foundation Laboratories, Weill Cornell Medicine, New York, NY, United States; ^6^Operative Unit of Pathology, S. Orsola Malpighi Hospital, Bologna, Italy; ^7^Department of Medical & Surgical Sciences, University of Bologna, Bologna, Italy; ^8^Biosciences Laboratory, Istituto Scientifico Romagnolo per lo Studio e la Cura dei Tumori (IRST), IRCCS, Meldola, Italy; ^9^Department of Medical Physics, S. Orsola-Malpighi University Hospital, Bologna, Italy; ^10^Interdepartimental Centre L. Galvani (CIG), University of Bologna, Bologna, Italy

**Keywords:** Human Papillomavirus (HPV), extracellular vesicles (EVs), triple negative BC, stromal cells, circulating HPV DNA

## Abstract

A causal link between Human Papillomavirus (HPV) and breast cancer (BC) remains controversial. In spite of this, the observation that HPV DNA is over-represented in the Triple Negative (TN) BC has been reported. Here we remark the high prevalence of HPV DNA (44.4%) in aggressive BC subtypes (TN and HER2+) in a population of 273 Italian women and we convey the presence of HPV DNA in the epithelial and stromal compartments by *in situ hybridization*. As previously reported, we also found that serum derived-extracellular vesicles (EVs) from BC affected patients contain HPV DNA. Interestingly, in one TNBC patient, the same HPV DNA type was detected in the serum-derived EVs, cervical and BC tissue samples. Then, we report that HPV DNA can be transferred by EVs to recipient BC stromal cells that show an activated phenotype (e.g., CD44, IL6 expression) and an enhanced capability to sustain mammospheres (MS) formation. These data suggest that HPV DNA vehiculated by EVs is a potential trigger for BC niche aggressiveness.

## Introduction

Human Papillomavirus (HPV) is a common pathogen in oropharyngeal and ano-genital cancers (1). The oncogenic potential of HPV resides on its capacity to interfere with some important regulators of the cell cycle via its oncogenic proteins E6 and E7. HPV has also been reported in a variety of cancers i.e., glioblastoma, colorectal, lung and breast cancers (2–5), but its pathogenic role remains controversial. Globally, breast cancer (BC) is the most frequent cancer among women (6). Some authors reported an increased risk of BC incidence among patients with a previous cervical dysplasia, suggesting an interplay between cervical infection and the development of BC (7). In the past decades, a wealth of studies have analyzed the presence of HPV DNA in BC tissues with a prevalence rate ranging from 0 to 86%, discrepancy mainly attributed to methodological and geographical differences across studies (8, 9). Recent literature, using the more powerful next generation sequencing, don't give support to viral infection in disease causality of BC (10). On the other side, some authors observed an association between TNBC phenotype and HPV positivity (11, 12), leading to the hypothesis that an imbalance of the native and/or adaptive immunity could favor the infection in a specific anatomical area, as well as the activation of an uncontrolled proliferation cycle. An unsolved problem is the origin of HPV DNA spreading to the mammary gland. Some authors speculate an ascending pattern of dissemination (13). However, circulating HPV DNA may contribute to HPV DNA spreading in unconventional site of infection. At this regard, some authors reported circulating HPV DNA in the serum of patients with HPV-associated invasive cancer (10) and as marker for disease extent and recurrence (14). We also found HPV DNA in the serum derived-extracellular vesicles (EVs) of breast pathologies affected women as well as in patient with HPV DNA positive squamous cell carcinoma of the middle rectum (15, 16). EVs are a heterogeneous population of vesicles, which includes exosomes, microvesicles, and apoptotic bodies that differ in size and biophysical properties (17). EVs are released from different types of tissue, cells and biological fluids and contain nucleic acids, proteins, non-coding RNAs and viral nucleic acids (18–22). Interestingly, exosomes and EVs have been implicated in HPV transmission and carcinogenesis (23–29). Moreover, some authors reported how stress condition and DNA damage induced by radiation therapy, can influence stromal compartment and the uptake of EVs (30, 31). Here, we report that HPV DNA is associated with aggressive BC phenotypes and that HPV DNA is present in serum derived-EVs of TNBC affected patients. Finally, we showed that EVs transfer HPV DNA to BC stromal cells, which acquire an inflammatory activated phenotype.

## Materials and Methods

### Formalin Fixed Paraffin-Embedded Breast Cancer (FFPE BC) Tissues Collection and Molecular Characterization

BC tissues (*n* = 273) were collected and archived at Sant'Orsola Malpighi Hospital, Bologna, Italy, by the Breast Cancer and Pathology Units, respectively. This study was approved by the local ethics committee (number 145/2015/U/Sper) and signed informed consent was obtained from all the patients enrolled. To define invasive carcinoma bioprofile, sections were treated in an automated immunostainer (Benchmark Ultra, Ventana Diagnostic Systems, USA) and immunostained using anti-ER (clone SP1), anti-PR (clone 1E2), anti-Ki67 (clone 30-9), anti-Her2 (clone 4B5) pre-diluted monoclonal antibodies all from Ventana. Sections were retrieved using UltraCC1 Tris-HCl buffer. The immunological reaction was visualized using the OptiView DAB Detection system (ER, PR, Ki67) or UltraView DAB Detection system (Her-2). Sections were counterstained on board with Hematoxylin II and Bluing reagents (Ventana Diagnostic Systems, USA). Immunostaining for ER, PR, and Ki67 was quantified using image cytometry with the IMAGE ProPlus 5.1 software (Media Cybernetics Inc., USA) and expressed as percentage of immunostained neoplastic cells. Her2 expression was evaluated following ASCO/CAP 2013 and 2018 recommendations and classified according to the Score 0/1+/2+/3+ method. Luminal cases were classified as Luminal A or Luminal B following the St. Gallen 2013-15 consensus recommendations, in particular we considered 20% as cut-off value.

### HPV DNA Chromogenic *in situ* Hybridization (CISH)

CISH was performed by the ZytoFast®Plus CISH Implementation Kit HRP-DAB (ZytoVision, Bio-Optica, Milan, Italy) using the ZytoFast HPV type 16/18 Probe digoxigenin-labeled probes according to manufacturer's protocol in order to detect HPV 16 and 18 in formalin fixed paraffin embedded BC and stromal compartment. HeLa and CaSki pellets were formalin fixed and paraffin embedded and were used as positive controls. As negative control we used HPV DNA negative cell lines (MCF7). Briefly, 20 × 10^6^ cells were centrifuged at 3,000 g for 10 min and resuspended in a small volume of PBS and mixed with agar. Then cells were fixed in formalin. We also used the ZytoFast DNA (-) Control Probe for assessing the unspecific background staining in formalin-fixed, paraffin embedded tissue or cells by chromogenic *in situ* hybridization (ZytoVision, Bio-Optica, Milan, Italy).

### Isolation of Breast Cancer Derived-Fibroblasts (BC DFs)

BC DFs (*n* = 20), were obtained from Breast Cancer Unit, Sant' Orsola Malpighi Hospital, with approval of the internal local ethics committee (006/2012/U/Tess; 145/2015/U/Sper) and upon the patient's written informed consent. Tissues samples were minced with scalpels in a tissue culture dish and then enzymatically dissociated in 5 mL of mammary epithelial growth medium (Cambrex, Milan, Italy) supplemented with 2% bovine serum albumin (Fraction V, Fisher Scientific), 10 ng/mL cholera toxin, 300 units/mL collagenase (Invitrogen, Milan, Italy), and 100 units/mL hyaluronidase (Calbiochem, Milan, Italy) at 37°C for 18 h. On the second day, the suspension was centrifuged at 80 × *g* for 4 min to separate the epithelial and fibroblast cells. Fibroblast cells were pelleted by centrifugation at 100 × *g* for 10 min followed by two washes with DMEM/F12 medium. The cell pellet was resuspended in DMEM/F12 medium supplemented with 5% FBS (Invitrogen, Milan, Italy) and 5 μg/mL insulin and plated in 25 cm^2^ tissue culture flasks. The cultures were incubated for 2–3 days at 37°C at 5% CO_2_. All the samples were stored at −80°C until use.

### Isolation of EVs From Serum Samples of BC Affected Patients

Serum specimens (*n* = 59), cervical cytological scrapes (*n* = 6) and TNBC tissues (*n* = 6), were collected from the Breast Cancer Unit, Sant'Orsola Malpighi Hospital, Bologna (Italy), from BC affected patients. This study was approved by the local ethics committee (145/2015/U/Sper) and patient's written informed consent was obtained. EVs were isolated from patients's serum specimens as reported by King et al. (32). Briefly, the serum specimens were centrifuged at 500 *g* for 10 min, at 18,000 g for 30 min and at 100,000 g for 120 min twice, to obtain the EVs pellet. The pellet was resuspended in 50 μL of PBS and was DNAseI digested (DNase I RNase-free 1 U/μL, Thermo Fisher, Milan, Italy) before nucleic acid extraction. EVs were counted by using the NS500 nanoparticle characterization system (Nanosight Malvern Instruments).

### HPV DNA Detection

DNA extraction from three 10 μm thick formalin fixed paraffin-embedded (FFPE) BC tissues was performed using NucleoSpin DNA FFPE Tissue kit (Macherey-Nagel, Milan, Italy). DNA extraction from BC DFs, CaSki derived-EVs, serum derived-EVs and from 6 TNBC affected patients specimens were performed by NucleoSpin Tissue kit (Macherey-Nagel, Milan, Italy). DNA concentration was measured by Thermo Scientific NanoDrop™ 1000 Spectrophotometer and stored at −20°C until use. HPV DNA detection in 273 BC tissues and 59 serum derived-EVs, was performed by Mass spectrometry assay (Mass Array Platform, Agena Bioscience, Hamburg, Germany), as previously described (15, 33). Briefly, this method is able to simultaneously detect and type 16 HPV DNA types (HPV16, 18, 31, 33, 35, 39, 45, 51, 52, 53, 56, 58, 59, 66, 68, and 73) in a single well. The viral DNA is amplified by a multiplex PCR with type-specific primers and the amplified product is extended at its 3′ terminal base with type specific primers. This method was as sensitive to detect up to 100 copies/reaction of HPV 16 and 18. HPV DNA detection in BC DFs and in the 6 TNBC affected patients specimens was performed by conventional PCR with MY09/11 and E6 HPV16 primer set on T100 Thermal cycler Bio-Rad (Table 1). Each amplification reaction was performed by GoTaq®Flexi DNA Polymerase kit (Promega, Milan, Italy) following the manufacturer's instruction. Amplified products were detected on 1.8% agarose gel by using ChemiDoc™ XRS+ System (Bio-Rad). Then, sequencing (GATC Biotech DNA Sequencing) of purified products was performed and nucleotide sequences were edited using the programme BioEdit Sequence Alignement. The sequences were compared with those deposited in the GenBank Database.

**Table 1:** Primers used for mRNA and DNA analysis by PCR assays.

**Target**	**Sequence (5^′^−3^′^)**	**NT position**	**Length (bps)**	**Assay**
L1 HPV DNA(MY09/11)	CGTCCMARRGGAWACTGATC^a^	N.A.	N.A.	PCR
	GCMCAGGGWCATAAYAATGG^a^	N.A.		
E6 HPV16 DNA	CAACAGTTACTGCGACGTGAG	206^b^	349	PCR
	GCTGGGTTTCTCTACGTGTTC	554^b^		
E7 HPV16 DNA	CAACTGATCTCTACTGTTATGAGCAA^c^	617^b^	73	Real-time and Digital PCR
	CCAGCTGGACCATCTATTTCA^c^	689^b^		
E1 HPV16 DNA	Taqman probe (Vi03453396_s1, Life Technologies)	N.A.	153	Real-time and Digital PCR
IL6 RNA	Taqman probe (Hs00985639_m1, Life Technologies)	N.A.	70	Real-time PCR
CD44 RNA	Taqman probe (Hs01075861_m1, Life technologies)	N.A.	66	Real-time PCR
Cyclin-D1 RNA	ACAAACAGATCATCCGCAAACAC	730^d^	144	Real-time PCR
	TGTTGGGGCTCCTCAGGTTC	873^d^		
c-Myc RNA	CTCTGACCTTTTGCCAGGAG	2036^e^	248	Real-time PCR
	CCTACCCTCTCAACGACAGC	1789^e^		
ACTB DNA	CCACACTGTGCCCATCTACG	1358^f^	98	Real-time PCR
	AGGATCTTCATGAGGTAGTCAGTCA	1456^f^		
ACTB DNA	Taqman probe (Hs03023880_g1, Life Technologies)	N.A.	139	Real-time PCR
GUSB RNA	Taqman probe (Hs99999908_m1, Life Technologies)	N.A.	81	Real-time PCR

*N.A. not available*.

a *Degenerate nucleotides of primers: W = T, C; Y = C, T; R = A,G; M = A,C*.

b *NCBI Reference Sequence HPV16: NC_001526.4*.

c *LNA Probe #63 (seq. AGGAGGAG),UPL Roche*.

d *NCBI Reference Sequence Cyc-d1: NM_053056.2*.

e *NCBI Reference Sequence C-Myc: NM_001354870.1*.

f *NCBI Reference Sequence ACTB: NC_000007.14*.

### Next Generation Sequencing (NGS)

NGS approach was used to analyze total DNA from serum derived-EVs of the patient with cervix, blood and BC coinfection of the same HPV type, namely HPV53. We amplify total DNA by modified degenerate FAP59/64 primers: 5′- CGTATCGCCTCCCTCGCGCCA-TCAGACGAGTGCGTTAACWGTIGGICAYCCWTATT-3′ and 5′- CTATGCGCCTTGCCAGCCCGCTCAGACGAGTGCGTCCWATATCWVHCATITCICCATC-3′ (W = T, C; I = inosine; Y = C, T; B = G, C, T; H = A, C, T; V = A, C, G) (34), which are able to amplify a broad spectrum of mucosal and cutaneous Papilloma Viruses (PVs). PCR was carried out on T100 Thermal cycler (Bio-Rad, Milan, Italy) using the following parameters: 10 min at 94°C and then 45 cycles of 30 s at 94°C, 30 s at 50°C and 60 s at 72°C. Then sequencing reaction was performed on Roche 454 Instrument following the manufacturer's instruction. PRINSEQ was used to filter, reformat, and trim sequence data (http://prinseq.sourceforge.net/) (35), the bases that fell below the PHRED score of 20 have been removed, reads with a length ≥20 bp were selected. Then raw files were analyzed using QIIME (v. 1.4.0-dev) (36) that was used to assign the taxonomy of each sequence (code “assign_taxonomy.py”), considering all the complete HPV genomes from Papilloma Virus Episteme web-based tool (humans and non-humans) (37).

### Isolation of Extracellular Vesicles (EVs) From Culture Media of CaSki Cells

HPV DNA positive cancer cell lines (CaSki) were purchased from the American Type Culture Collection (ATCC). CaSki cells were established from a metastasis of a cervical cancer and are reported to contain about 600 copies per cell of integrated HPV16 DNA. CaSki cell lines were cultured in RPMI-1640, supplemented with 10% fetal bovine serum (Euroclone, Milan, Italy), 1% glutamine and 1% penicillin/streptomycin at 37°C in 5% CO_2_ humidified atmosphere. EVs were isolated from 80 ml of HPV DNA positive CaSki cell lines supernatant, maintained in serum-free medium for 48 h at 37°C in 5% CO_2_ humidified chamber as reported previously.

### HPV DNA Quantification

HPV DNA quantification in CasKi derived-EVs was performed by Taqman assay (TaqMan Universal PCR Master Mix, Thermo Fisher Scientific, Milan, Italy) and SYBR Green (SYBR® Select Master Mix for CFX, Life Technologies, Milan, Italy) following the manufacturer's instruction, with E1, E7, and E6 HPV16 primer pairs (Table 1). The beta-Actin was used as endogenous controls. The data were analyzed by using the 2^−ΔΔ*CT*^ method. Each sample was analyzed in triplicate. Digital PCR was performed on QuantStudio® 3D Digital PCR System (Life Technologies, Milan, Italy), with E7 and E1 HPV16 primer pairs (Table 1) by Taqman assay (Life Technologies, Milan, Italy), following the manufacturer's instruction.

### Nanosight and FACS Analysis of EVs

EVs were diluted in 1 ml of PBS EVs-free, loaded into the sample chamber of an LM10 unit (Nanosight, Malvern, UK) and three videos of either 30 or 60 s were recorded of each sample. This analysis allows to identify the vesicles size's distribution profile and their concentration (particles/ml). EVs surface antigens were investigated with the MACSPlex Exosome kit (cat. no.130-108-813, Miltenyi Biotec GmbH, Gladbach, Germany) (38). MACSPlex Exosome kit detects 37 surface markers, including: CD9, CD81, CD63, cell adhesion, migration, proliferation and immune cells markers. Briefly, after isolation, EVs were diluted in MACSPlex buffer and stained according to manufacturer's instructions and samples were analyzed by FACSCanto flow cytometer (Beckton Dickinson). At least 10,000 events per sample were recorded and data were analyzed with FACSDiva software. The median fluorescence values plotted in the graph were background corrected and normalized on CD63/81/9 median signal intensity. Negative values were excluded from the plot.

### EVs Administration to BC DFs

100,000 BC DFs were seeded in DMEM medium supplemented with 20% FBS, 1% glutamine and 1% penicillin/streptomycin in a 6 well plate and incubated at 37°C in 5% CO_2_ humidified atmosphere. After 24 h, BC DFs were grown in DMEM serum-free and then were exposed to a single radiation dose of 7.5 Gy using a photon beam generated by a roentgen with 180 kV and filtration 0.35 Cu−1.5 Al. For the setup we used a base of 8 cm of solid water and a field 20 × 20 cm^2^ with dose rate of 62 CGy/m. An amount of 2 × 10^10^ CaSki-derived EVs in 1 ml volume of medium was administered at time 0 to irradiated 100,000 BC DFs, the medium was removed at 24 h post irradiation and substituted at 24, 48, and 72 h with fresh DMEM without HPV DNA positive EVs. At day 4, the medium was collected and 1 ml volume was administered to 20,000 MCF7 cells cultured in 3D model in low attachments plates to perform MCF7 derived-MS assay.

### RNA Extraction and Gene Expression Analysis

RNA extraction was performed on EVs-administered BC DFs by TRIzol reagent (Life Technologies, Milan, Italy). An amount of 0.1–1 μg of total RNA was retro-transcribed by the Master RT plus PCR system kit (LifeTechnologies, Milan, Italy). RNA concentration was measured by Thermo Scientific NanoDrop™ 1000 Spectrophotometer and stored at −80°C until use. cDNA was amplified by SYBR Green (SYBR® Select Master Mix for CFX, Life Technologies, Milan, Italy) and Taqman assay (TaqMan Universal PCR Master Mix, Thermo Fisher Scientific, Milan, Italy), following the manufacturer's instruction, with IL6, CD44, c-Myc, and Cyclin-D1 primers (Table 1). Real time PCR was performed on Mx3000P Stratagene instrument (Agilent Technologies). The beta-glucuronidase gene, were used as endogenous controls. The data were analyzed by using the 2^−ΔΔ*CT*^ method. Each sample was analyzed in triplicate.

### MCF7 and MDA-MB-231 Derived-MS Assay

The MCF7 and MDA-MB-231 human BC cell lines were purchased by the American Type Culture Collection (ATCC). MCF7 and MDA-MB-231 cells were cultured in RPMI-1640 and DMEM respectively, supplemented with 10% fetal bovine serum (Euroclone, Milan, Italy), 1% glutamine and 1% penicillin/streptomycin at 37°C in 5% CO_2_ humidified atmosphere. An amount of 20,000 BC cells were resuspended in medium serum-free and filtered through a 40 μm nylon mesh (Becton Dickinson, Franklin Lakes, NJ) before being suspended in 1.5 cm^2^ low attachment wells (Becton Dickinson, Franklin Lakes, NJ) with 1 ml of medium serum-free, 1% penicillin/streptomycin. BC cells were incubated at 37°C in 5% CO_2_ humidified atmosphere. MCF7 and MDA-MB-231 derived-MS were counted after 6 days of incubation as average number of cells per field of view with OLYMPUS CKX41 microscopy.

### Soft Agar Assay

The Soft Agar Assay is an anchorage independent growth assay in soft agar, which is considered the most stringent assay for detecting malignant transformation of cells. In brief, 5,000 cells were cultured on a 24 well plate containing 1% base agar and 0.35% top agar in the DMEM medium and incubated at 37°C for 15 days. Plates were stained with 0.005% crystal violet + methanol for 1 h. Colonies were acquired with ChemiDoc™ XRS+ System (Bio-Rad) and counted using ImageJ software.

### Clonogenic Assay

Clonogenic Assay or Colony Formation Assay is an *in vitro* cell survival assay based on the ability of a single cell to grow into a colony. This assay measure cells survival skills based on the number of colonies formed after low density seeding. In brief, 5000 cells were cultured on a 24 well plate at 37°C for 15 days. After 15 days medium was removed and cells were rinsed with PBS. Fixation and staining of clones were done with a mixture of 0.5% crystal violet in 50/50 methanol/water for 30 min. Plates were rinsed with water and left for drying at room temperature. Counting of clones was done on the following day. Clones were acquired with ChemiDoc™ XRS+ System (Bio-Rad) and counted with ImageJ software.

### Statistical Analysis

Statistical analysis was performed by SPSS software (SPSS Incorporation). Chi square test was calculated with Monte Carlo method. The exact *p*-value was calculated by un-paired *t*-test (two groups comparisons) and one-way ANOVA (>2 groups comparisons). *Post-hoc t*-test values were corrected for multiple comparisons according to *Bonferroni* correction. The statistical analysis was performed with Graph-pad Prism 6 software.

## Results

### Association Between HPV DNA With TNBC and BC Malignant Features

**Figure 1:**
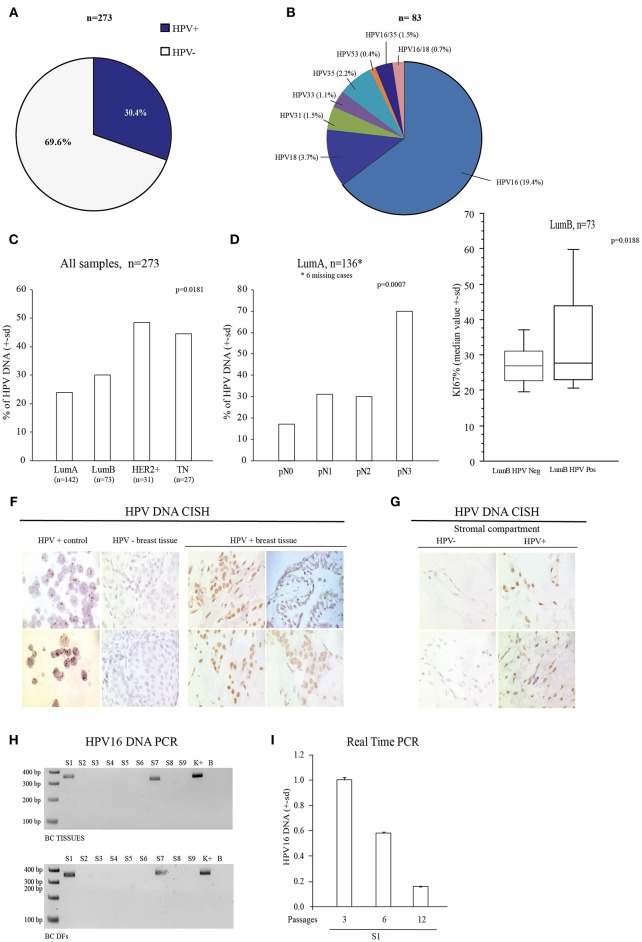
Association between HPV DNA and Breast Cancer (BC). **(A)** HPV DNA prevalence in 273 FFPE breast cancer (BC) tissues. **(B)** Percentage of HPV genotypes in the 83 FFPE HPV positive BC tissues. **(C)** Association of HPV DNA in the different BC subtypes (Lum A and B, HER2+ and TN), *p*-value = 0.0181. **(D)** Association between HPV DNA and extent of lymph nodes invasion in LumA BC (*n* = 136, missing 6 cases), *p*-value = 0.0007. **(E)** Association of Ki67 in HPV DNA positive LumB BC (*n* = 73), *p*-value = 0.0188. **(F)** HPV DNA Chromogenic *in situ* Hybridization assay (CISH), in BC tissues. HPV DNA negative BC tissues, HeLa and CaSki cell lines are reported as controls. **(G)** HPV DNA CISH assay in stromal compartment of BC tissues. **(H)** PCR analysis of E6 HPV16 DNA in 9 BC tissues and 9 corresponding isolated BC DFs (S1-S9 samples). **(I)** Real Time PCR analysis of HPV16 E7 DNA in HPV16 positive *ex vivo* isolated S1 BC DF cultured for at least 12 passages. This sample are used as reference to detect HPV16 DNA. Data are presented as mean ± s.d.; p refers to *t*-test; *p < 0.0001.

This study started by investigating the presence of sixteen high-risk mucosal HPV DNA types by MALDI-TOF technique (33) to prove the association of HPV with the TNBC subtype. Purposely, we retrospectively assessed HPV DNA in 273 FFPE BC tissues and we found that 83 samples (30.4%) were HPV DNA positive (Figure 1A) and that HPV16 was the most prevalent HPV type (Figure 1B). We found that HPV DNA was over-represented in the TN subtype (12/27, 44.4%), in aggressive HER2+ BC (15/31, 48.4 %) compared to Luminal A (LumA) (34/142, 23.9%) and Luminal B (LumB) ones (22/73, 30.1%) (Monte Carlo *X* square test: *P* = 0.0181, Figure 1C). These data support the previously association between TN subtype and HPV DNA (12). Notably, in LumA cases HPV DNA presence was proportionally related to the extent of lymph node invasion (pN1 = 31%; pN2 = 30%; pN3 = 70%—Monte Carlo X^2^ square test: *P* = 0.0007, Figure 1D, Table 2, Supplementary Table 1). HPV DNA was also associated with the highest proliferation rate (Ki67), only in the LumB subtype (*p* = 0.0188) (Figure 1E, Supplementary Table 2). We also assessed the presence of HPV DNA in BC specimens by Colorimetric *in situ* Hybridization (CISH) (Figure 1F). We observed the presence of HPV DNA in BC epithelial cells, in the stromal compartment and in endothelial cells (Figure 1G). To confirm the presence of HPV DNA specific signal in stromal compartment, we assessed HPV DNA by PCR in *ex vivo* cultured BC derived-fibroblast (DFs). We found the presence of HPV16 DNA in 2 out of 9 BC DFs, one from an HPV16 DNA positive TNBC and one from an HPV16 DNA positive LumB cancer (Figure 1H). BC DF from the HPV16 DNA positive TNBC, still showed the presence of HPV DNA, after 12 *in vitro* passages (Figure 1I). These data support the presence of HPV DNA in the breast tissue and encouraged us to investigate how HPV may spread to the mammary gland and how the presence/persistence of HPV DNA in fibroblasts may influence the tumorigenic process.

**Table 2:** Clinical pathological variables of 273 BC samples.

**Subtype/Stage/Molecular marker**	**HPV DNA+**	**HPV DNA-**
T1 (*n* = 181^*^)	53	128
T2 (*n* = 70)	23	47
T3 (*n* = 9)	2	7
T4 (*n* = 13)	5	8
N0 (*n* = 150^°^)	36	114
N1 (*n* = 59^°^)	20	39
N2 (*n* = 24^°^)	9	15
N3 (*n* = 29^°^)	16	13
G1 (*n* = 41^ª^)	8	33
G2 (*n* = 134^ª^)	40	94
G3 (*n* = 77^ª^)	28	49

* missing 3 cases;

° missing 11 cases;

ª *missing 21 cases; BC, Breast Cancer; T1-4, Size of the tumor; N0-3, Lymph node status; G, Grading*.

### HPV DNA in Circulating EVs of TNBC Patients

**Figure 2:**
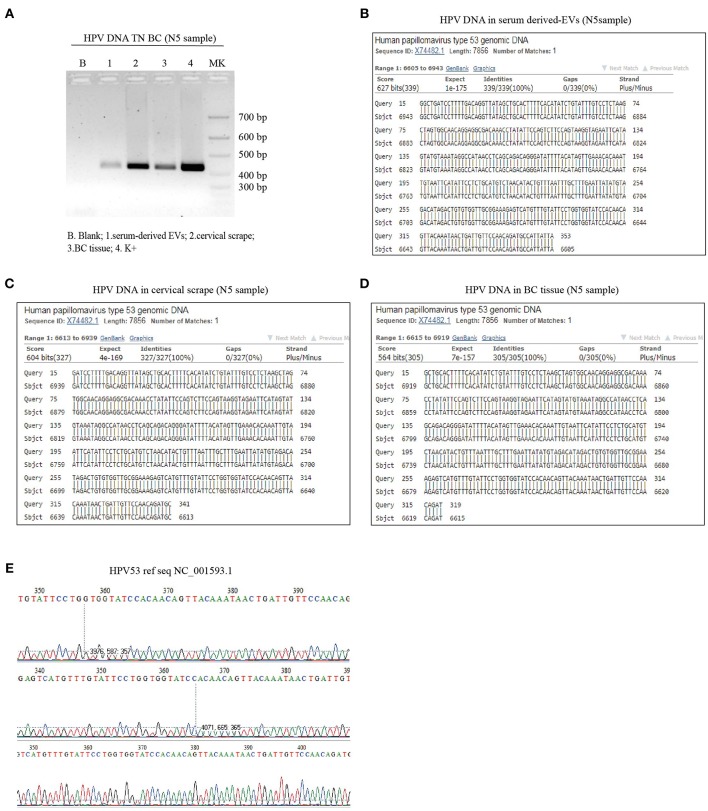
HPV DNA assessment in cervical scrape, serum derived-EV and BC tissues of TNBC affected patients. **(A)** PCR analysis of cervical scrape, serum derived-EVs and BC specimens with MY09/11 consensus primer: B-blank, 1-serum derived-EVs, 2-servical cytological specimen, 3-BC tissue. **(B–E)** Sequencing of PCR product obtained from serum-derived EVs **(B)**, cervical scrape **(C)**, and BC tissue **(D)**. GenBank database are used as reference sequence of HPV53 genotype (NC_001593.1) **(E)**.

We previously demonstrated that circulating HPV DNA can be found in serum derived-extracellular vesicles (EVs) in women with breast pathologies as well as in patient with HPV DNA positive squamous cell carcinoma of the middle rectum (15, 16). Here, as described in the first part of the study approved by the local ethics committee (145/2015/U/Sper), we enrolled 59 BC affected-patients and we searched for the presence of HPV DNA in the serum derived-EVs. We found the presence of HPV DNA in 7 patients (11.9%): 3 out of 23 LumA (13.0%), 2 out of 8 HER2+ (25%) and 2 out of 6 TNBC (33.3%) (Table 3). As delineated in the second phase of the study, of all the TNBC patients, whose EVs were available, we collected cervical scrapes and FFPE BC tissues, with the aim to investigate HPV DNA dissemination by EVs from a primary site of infection to distant sites, e.g., breast tissues. Three out of 6 TNBC tissues (50%) were positive for HPV DNA (Table 4). In particular, in one patient HPV16 DNA was detected both in cervical scrape and BC tissue sample. In another one, we found HPV6 DNA, in cervical specimen and serum derived-EVs (Table 4). In the last patient, we detected the same HPV DNA strain (HPV53) in cervical scrape, in serum-derived EVs and in the BC tissue (Figures 2A–E). We also confirmed the presence of high levels of HPV53 (12732 reads, 99.72%) in the serum-derived EVs, by NGS, with a primer set able to amplify both mucosal and cutaneous HPV types. Even if the distribution of HPV 53 is more common in CIN1 (cervical intraepithelial neoplasia grade 1) and normal cases, this genotype is considered an high risk HPV (39). These data support our hypothesis that HPV DNA can be vehiculated by EVs from primary sites of infection, e.g., cervical tissues, to distant body districts, such as the mammary gland, where HPV DNA can be found even if cells are not permissive for viral replication.

**Table 3:** HPV DNA assessment in 59 serum derived-EVs.

**BC subtype**	**N**	**HPV DNA positive specimens (%)**	**HPV DNA negative specimens (%)**	**HPV genotypes**
LumA	23	3 (13.0%)	20 (87%)	16, 16/31, 16/33
LumB	22	0 (0.0%)	22 (100.0%)	-
HER2+	8	2 (25.0%)	6 (75.0%)	31,18
TN	6	2 (33.3%)	4 (66.7%)	53, 6
	59	7 (11.9%)	52 (88.1%)	-

*BC, Breast Cancer; LumA, Luminal A; LumB, Luminal B; HER2+, Human Epidermal growth factor Receptor 2; TN, Triple Negative*.

**Table 4:** HPV DNA assessment in cervical scrapes, serum derived-EVs and BC tissues from 6 TNBC affected patients.

**Patient**	**HPV DNA in cervical scrape**	**HPV DNA in serum-derived EVs**	**HPV DNA in TNBC tissue**
N1	Neg	Neg	Neg
N2	Neg	Neg	HPV16
N3	Neg	Neg	Neg
N4	Neg (HPV16 past infection and CIN1 lesion)	Neg	HPV16
N5	HPV53	HPV53	HPV53
N6	HPV6	HPV6	Neg

*EVs, extracellular vesicles; TNBC, triple negative breast cancer; CIN1, cervical intraepithelial lesion grade 1*.

### HPV DNA Transfer to Fibroblasts Is Mediated by EVs

**Figure 3:**
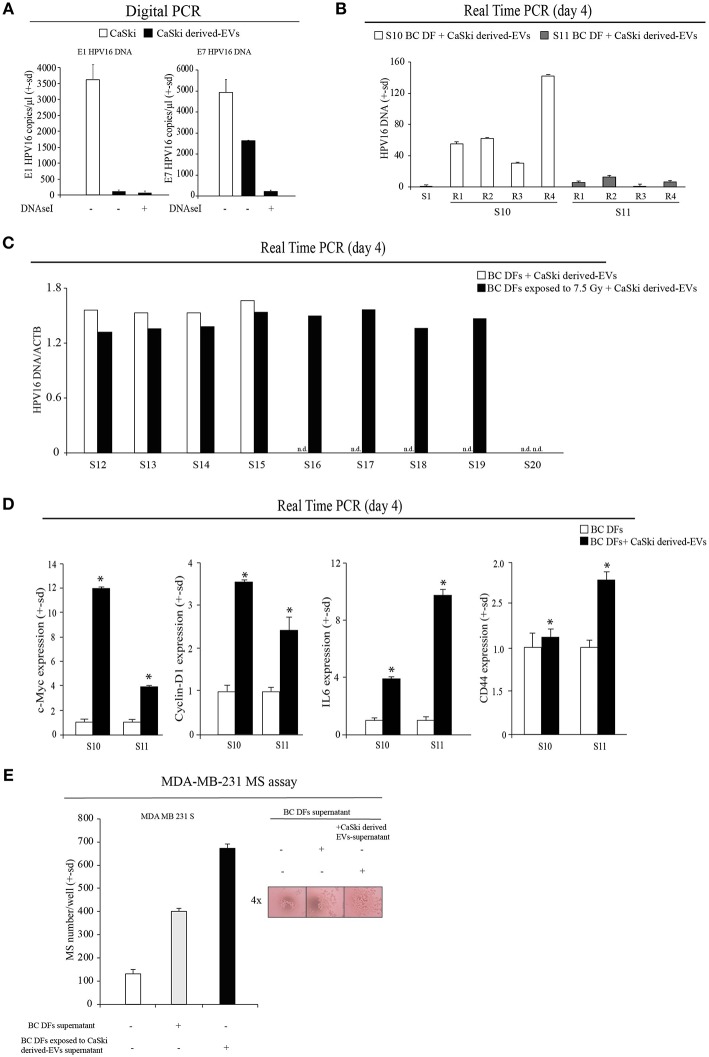
HPV DNA transfer to the BC niche is mediated by EVs. **(A)** Digital PCR analysis of HPV DNA content (E1 and E7) in CaSki cells and CaSki derived-EVs (copies/ul). **(B)** Real Time PCR analysis of HPV16 DNA content (E7) in BC DFs exposed to CaSki derived-EVs for 4 days (S10-S11 samples). The specimens were analyzed in quadruplicate (R1-R4). The data were normalized on HPV DNA content of the *ex vivo* isolated S1 BC DF sample. **(C)** Real Time PCR analysis of HPV DNA (E7) in 9 BC DFs exposed to a single dose of 7.5 Gy of X-Rays and to CaSki derived-EVs for 4 days (S12-S20 samples) (n.d., not detected). **(D)** Real Time PCR analysis of c-Myc, Cyclin-D1, IL6 and CD44 mRNAs in BC DFs of exposed to CaSki derived-EVs upon 4 days (S10-S11 samples). **(E)** MS assay of MDA-MB-231 cells administered with the supernatant of BC DFs for 6 days, compared to control. MS were counted and represented as mean ± s.d. p refers to *t*-test; *p < 0.0001.

**Figure 4:**
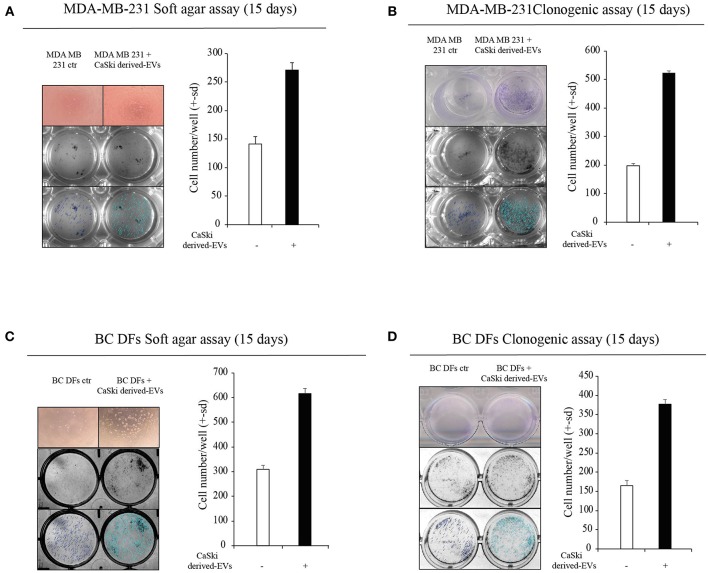
Effects of HPV DNA+ EVs transfer on epithelial and stromal compartments. **(A,B)** Soft-agar and Clonogenic Assays on MDA-MB-231 after 15 days of exposure to CaSki-derived EVs, versus control (three independent experiments). Data are presented as mean ± s.d. **(C,D)** Soft-agar and Clonogenic Assays on BC DFs after 15 days of exposure to CaSki-derived EVs, versus control (three independent experiments). Data are presented as mean ± s.d.; p refers to *t*-test; *p < 0.0001.

We then tested the hypothesis that EVs can transfer HPV DNA to negative recipient cells. Purposely, we isolated EVs from the supernatant of CaSki cell line, which contain integrated HPV16 DNA and not complete viral particles. EVs were characterized by Nanosight, and FACS analysis (Supplementary Figures 1A,B). Before DNA extraction, EVs were treated by DNAseI to avoid cell free DNA contamination and were assessed by HPV16 specific Digital PCR assay (Figure 3A). Then, we tested the capability of CaSki-derived HPV DNA + EVs to transfer HPV DNA to *ex vivo* isolated BC DFs. At this purpose we administered an arbitrary fixed amount of 2^*^10^10^ EVs to HPV negative BC DFs and we observed different capability to uptake and to retain HPV DNA from EVs after 4 days of exposure (Figure 3B). Interestingly, it has been reported that EVs uptake were increased when recipient cells are exposed to radiation (30). Purposely, we first exposed 9 HPV negative BC DFs to a single dose of 7.5 X-Ray radiation and then we administered them 2^*^10^10^ CaSki derived-HPV DNA+EVs. As previously reported, we found heterogeneous capability to retain HPV DNA along with BC DFs strains. In particular, after X-Ray single dose exposure, all but one BC DFs strains show detectable level of HPV DNA compared to 4 out of 9 BC DFs without X-Ray exposure (Figure 3C). We also analyzed the effects of HPV DNA+ EVs uptake in BC DFs by analyzing some proliferation and stromal markers. We found an increased level of c-Myc and Cyclin-D1 expression in exposed DFs, compared to not exposed cells, as well as an increased level of stromal activation markers, e.g., IL6 and CD44 (Figure 3D). Accordingly, the supernatant of BC DFs exposed to HPV DNA+ EVs was more capable to elicit MDA-MB-231 and MCF7 derived-MS formation than control (Figure 3E, Supplementary Figure 1C). Accordingly to the finding that HPV DNA is more represented in TNBC, we administered HPV DNA+ EVs on MDA-MB-231. We observed an enhanced cellular invasion and proliferation by Soft-Agar and Clonogenic Assays (Figures 4A,B). HPV DNA+ EVs contribution in the cellular proliferation and aggressiveness were also confirmed in BC DFs exposed to CaSki derived-EVs (Figures 4C,D). These results showed that HPV positive EVs increased pro-tumorigenic activity in the BC niche.

## Discussion

Breast cancer is a frequent pathology in the female population, representing 24% of diagnosed pathologies and 15% of mortality (40). Despite HPV DNA has been largely investigated in breast cancer tissues, its role in breast pathology remains to be elucidated (8, 9). The data presented here report that HPV DNA is over-represented in the TNBC and HER2+ BC tissues than in the Luminal ones. Furthermore, among Luminal subtypes, HPV DNA was more prevalent in highly lymph-node-invasive LumA cancers tissues as well as among LumB tissues showing high levels of Ki67 expression. As a support of this conclusion is the finding that, according to recent literature, HPV DNA is more represented among TNBC tissues (11, 12). Overall, these data sustain the notion that HPV DNA is present in breast cancers with enhanced aggressive features. Nevertheless, we presume that HPV DNA is not *per se* sufficient to induce the carcinogenic process but, as environmental factor, it may contribute to delineate the BC tumorigenic phenotype. Interestingly, we observed the presence of HPV DNA not only in the epithelial compartment but also in the stromal one by *in situ hybridization*. We support this finding by analyzing *ex vivo*-cultured fibroblasts, derived from BC tissues, which showed the presence of HPV DNA, even after several *in vitro* culture passages. Notably, the presence of HPV DNA in human primary fibroblasts, that lack receptors for HPV, has been reported as mediated by apoptotic bodies derived from HPV DNA positive cell lines (23, 26, 28). In this regard, we previously demonstrated that HPV DNA may be vehiculated by circulating EVs in patients with breast pathologies and squamous cell carcinoma of the middle rectum (15, 16). We thus hypothesized that HPV DNA transfer to recipient cells may be supported by EVs. Here, we found the presence of HPV DNA in 7 out of 59 serum derived-EVs of BC affected patients. Two out of 7 were EVs from TNBC patients. Interestingly, in one of the two cases, the same HPV genotype, i.e., HPV53, was shared among the EVs, the breast tissue and the cervical specimen. HPV 53 is considered a high risk HPV, even if its distribution is more common in CIN1 (cervical intraepithelial neoplasia grade 1) and normal cervix vs. high-grade cervical lesions and cancer. In any case, the presence of an HPV DNA+ high-grade cervical lesion or cancer does not seem a prerequisite for HPV DNA shedding via EVs, in fact none of our 6 TNBC affected patients showed high grade cervical lesions. Furthermore, in 2 out of 3 HPV DNA + TNBC we did not observed the simultaneous presence of HPV in the BC tissue, cervix and EVs (see Table 4). In one case, the cervical infection has already been resolved and in the other one, we cannot exclude that an alternative primary site of infection has been involved in this dynamic process, e.g., the oropharyngeal site or the rectum. Eventually, it seems that our findings depicted a static picture of a long-lasting dynamic process that might involve the shedding of HPV DNA + EVs from a primary site of infection to distant districts, e.g., the breast tissue. This finding is reminiscent of the previous reported by Lawson et al. (7). On the basis of these observations, we tested *in vitro* the hypothesis that HPV DNA positive EVs are able to transfer their cargo to recipient cells. At this regard, we administered HPV DNA + EVs to BC DFs and we found heterogeneous but reproducible persistence of HPV DNA in recipient cells. Moreover, we found that the exposure of BC DFs to stress (single dose of 7.5 Gy of X-Rays) increased the capability to retain HPV DNA after exposure to HPV DNA + EVs. Hence, our data indicate that HPV DNA can be transferred to BC DFs by EVs and that this phenomenon is enhanced by cellular stress. It is worth noting that radiation exposure increase the uptake of EVs and the internalization of EVs carried genetic material (30, 31). In agreement with literature (24, 25, 29), we showed that the effect of HPV DNA + EVs transfer was to induce an activated phenotype in BC DFs, as demonstrated by the increased expression of fibroblast activation and proliferation markers. At this regard, we show that HPV DNA transfer to stromal cells may indirectly influence the aggressiveness of triple negative mammary epithelial cells by enhancing mammospheres formation, which represents an index of aggressiveness. Nevertheless, we can't exclude that HPV DNA + EVs might transfer other factors, simultaneously with HPV DNA, able to influence fibroblasts activation and mammospheres formation. Uptake of nucleic acids via EVs is a mechanism involved in different types of cancer (29, 41–45). In particular, it has been observed that the release of some microRNAs via EVs derived-fibroblast in breast cancer cells, was able to increase proliferation, EMT and the ability to form MS (43, 46, 47). Our data suggest that exposure to HPV DNA positive EVs or apoptotic bodies, see ref. (23, 26, 28), may be able to remodel the BC microenvironment by activating BC DFs (30, 44–46).

In our model, HPV DNA + EVs, produced from a primary site of infection, are able to transfer their content to recipient cells that lack HPV receptors, e.g., fibroblasts and BC epithelial cells. This transfer is not sufficient *per se* to promote BC but might contribute, as environmental factor, to confer aggressive feature to the BC. The uptake of HPV DNA + EVs in BC stromal cells may be modulated by inflammatory stimuli, such as exposure to X-Rays radiation, which may also increase the retention of HPV DNA in stromal cells. This persistence may promote an activation of the stromal compartment, which in turn promote an enhanced aggressiveness in the BC epithelial counterpart.

## Data Availability

All datasets for this study are included in the manuscript/Supplementary Files.

## Ethics Statement

This study was carried out in accordance with the recommendations of Sant'Orsola Malpighi Hospital Ethics Committee, Bologna, Italy, with written informed consent from all subjects. All subjects gave written informed consent in accordance with the Declaration of Helsinki. The protocols 145/2015/U/Sper and 006/2012/U/Tess were approved by Sant'Orsola Malpighi Hospital Ethics Committee, Bologna, Italy.

## Author Contributions

SD, MB, and MC conceived and developed the study. SD, GS, CC, CS, LG, FF, and MC performed experiments. FR and GC performed X-ray irradiation experiments. SD, GS, CC, CG, PG, MB, and MC, analyzed data. CC, DS, RS, and MT collected and provided patient samples. SD, GS, PS, MB, and MC wrote the paper. All the authors discussed the results and commented on the manuscript.

### Conflict of Interest Statement

The authors declare that the research was conducted in the absence of any commercial or financial relationships that could be construed as a potential conflict of interest.

## Supplementary Material

Data Sheet 1 Different grade of limph nodes invasion (pN0-pN3) in the 4 BC subtypes vs. HPV status (Supplementary Table 1). Mean values and standard deviations of the proliferation rate (Ki67) in the 4 BC subtypes vs. HPV status (Supplementary Table 2).

Data Sheet 2 Classification of the 273 breast cancer tissues according to molecular characterization and HPV assessment.

Supplementary Figure 1 **(A)** Nanosight analysis of CaSki derived-EVs size (140.2 nm) and concentrations (1.98 × 10^10^ particles/ml). **(B)** FACS analysis of CaSki derived-EVs by MACSPlex Exosome kit. **(C)** MS assay of MCF7 cells administered with the supernatant of BC DFs for 6 days, compared to control. MS were counted and represented as mean ± s.d.
