# Supplementary material for: HPV DNA Associates With Breast Cancer Malignancy and It Is Transferred to Breast Cancer Stromal Cells by Extracellular Vesicles
Source: Front Oncol. 2019 Sep 16;9:860. doi: 10.3389/fonc.2019.00860 (PMC6756191; doi:10.3389/fonc.2019.00860)
Supplement: Data Sheet 1 — Different grade of limph nodes invasion (pN0-pN3) in the 4 BC subtypes vs. HPV status (Supplementary Table 1). Mean values and standard deviations of the proliferation rate (Ki67) in the 4 BC subtypes vs. HPV status (Supplementary Table 2). [file Data_Sheet_1.docx]

**Supplementary Table 1: Different grade of limph nodes invasion (pN0-pN3) in the 4 BC subtypes versus HPV status**

| **Subtypes** | **pN0** | **pN1** | **pN2** | **pN3** | **Total** |
| --- | --- | --- | --- | --- | --- |
| **Her2 HPV Neg** | 10 | 4 | 1 | 1 | **16** |
| **Her2 HPV Pos (1 missing 1 case)** | 4 | 5 | 2 | 3 | **14** |
| **LumA HPV Neg (missing 6 cases)** | 72 | 20 | 7 | 3 | **102** |
| **LumA HPV Pos** | 15 | 9 | 3 | 7 | **34** |
| **LumB HPV Neg (missing 3 cases)** | 22 | 12 | 6 | 8 | **48** |
| **LumB HPV Pos (missing 1 case)** | 10 | 4 | 3 | 4 | **21** |
| **TN HPV Neg** | 10 | 3 | 1 | 1 | **15** |
| **TN HPV Pos** | 7 | 2 | 1 | 2 | **12** |
| **Total** | **150** | **59** | **24** | **29** | **262*** |

*missing 11 cases

**Supplementary Table 2: Mean values and standard deviations of the proliferation rate (Ki67) in the 4 BC subtypes versus HPV status**

| **Subtypes** | **Count** | **Mean** | **Std. Dev.** | **P-Value** |
| --- | --- | --- | --- | --- |
| **Her2 HPV Neg** | 16 | 32,206 | 17,275 | 0,3445 |
| **Her2 HPV Pos** | 15 | 27,167 | 11,015 |  |
| **LumA HPV Neg** | 108 | 9,405 | 4,905 | 0,9659 |
| **LumA HPV Pos** | 34 | 9,365 | 4,148 |  |
| **LumB HPV Neg** | 51 | 27,527 | 8,124 | 0,0188 |
| **LumB HPV Pos** | 22 | 34,186 | 15,531 |  |
| **TN HPV Neg** | 15 | 60,340 | 18,057 | 0,4861 |
| **TN HPV Pos** | 12 | 67,058 | 30,869 |  |
