# Supplementary figures and images for: HPV DNA Associates With Breast Cancer Malignancy and It Is Transferred to Breast Cancer Stromal Cells by Extracellular Vesicles
Source: Front Oncol. 2019 Sep 16;9:860. doi: 10.3389/fonc.2019.00860 (PMC6756191; doi:10.3389/fonc.2019.00860)

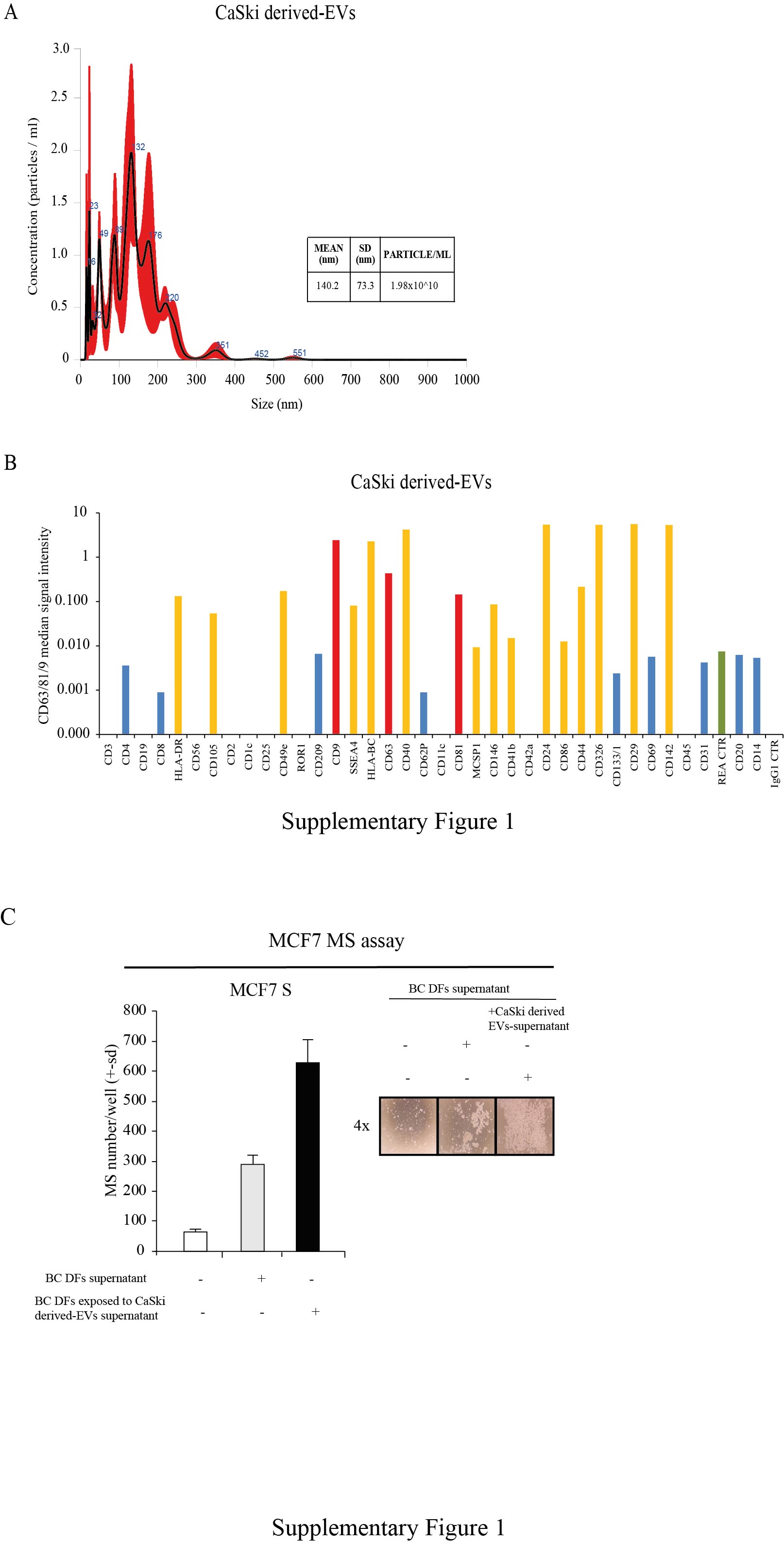

Supplement: Supplementary Figure 1 — (A) Nanosight analysis of CaSki derived-EVs size (140.2 nm) and concentrations (1.98 × 10^10^ particles/ml). (B) FACS analysis of CaSki derived-EVs by MACSPlex Exosome kit. (C) MS assay of MCF7 cells administered with the supernatant of BC DFs for 6 days, compared to control. MS were counted and represented as mean ± s.d. [file Image_1.JPEG]
